# Methyl 3-(4-fluoro­phen­yl)-1-methyl-1,2,3,3a,4,9b-hexa­hydro­chromeno[4,3-*b*]pyrrole-3a-carboxyl­ate

**DOI:** 10.1107/S1600536810054036

**Published:** 2011-01-12

**Authors:** G. Chitra Devi, Sundari Bhaskaran, G. Usha, G. Murugan, M. Bakthadoss

**Affiliations:** a Department of Physics, Queen Mary’s College (A), Chennai-4, Tamilnadu, India; b Department of Organic Chemistry, University of Madras, Guindy Campus, Chennai-25, Tamilnadu, India

## Abstract

In the title compound, C_20_H_20_FNO_3_, the pyrrolidine and benzopyran rings adopt half chair and twisted half chair conformations, respectively. The carboxyl­ate group is almost perpendicular to the pyran ring [89.4 (1)°].

## Related literature

Chromanone derivatives are used as inter­mediates in the synthesis of natural products calonlide (A) and inophyllum (B) (Ellis *et al.*, 1977 ▶), which have been suggested to have activity against anti-human immuno deficiency virus type 1 (HIV-1) (Hussain & Amir, 1986 ▶). Chromanone derivatives dilate the heart and act as remedies for angina pectoris, see: Hasegnaida (1967 ▶). Pyrrolidine derivatives possess anti-influenza (Stylianakis *et al.*, 2003 ▶) and anti-convulsant (Obniska *et al.*, 2002 ▶) activity. For related structures, see: Abdul Ajees *et al.* (2002 ▶); Usha *et al.* (2003 ▶). For asymmetry parameters, see: Nardelli (1983 ▶). 
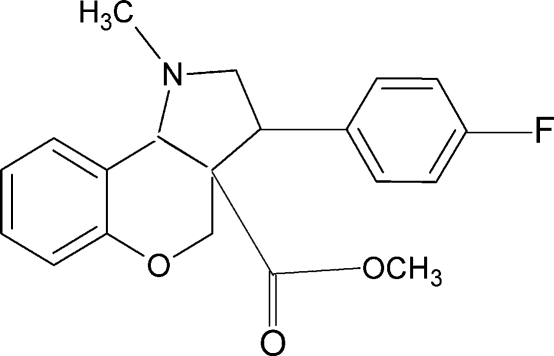


## Experimental

### 

#### Crystal data

C_20_H_20_FNO_3_
*M* *_r_* = 341.37
Monoclinic, 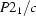

*a* = 10.4519 (4) Å
*b* = 20.6778 (8) Å
*c* = 7.8508 (3) Å
β = 91.535 (2)°
*V* = 1696.12 (11) Å^3^
*Z* = 4
Mo *K*α radiation
μ = 0.10 mm^−1^
*T* = 293 K
0.22 × 0.20 × 0.19 mm

#### Data collection

Bruker Kappa APEXII CCD diffractometer
16737 measured reflections
4237 independent reflections
2844 reflections with *I* > 2σ(*I*)
*R*_int_ = 0.035

#### Refinement

*R*[*F* ^2^ > 2σ(*F* ^2^)] = 0.045
*wR*(*F* ^2^) = 0.219
*S* = 0.81
4237 reflections
227 parameters
H-atom parameters constrained
Δρ_max_ = 0.24 e Å^−3^
Δρ_min_ = −0.18 e Å^−3^

### 

Data collection: *APEX2* (Bruker, 2004 ▶); cell refinement: *SAINT* (Bruker, 2004 ▶); data reduction: *SAINT* and *XPREP* (Bruker, 2004 ▶); program(s) used to solve structure: *SHELXS97*(Sheldrick, 2008 ▶); program(s) used to refine structure: *SHELXL97* (Sheldrick, 2008 ▶); molecular graphics: *ORTEP-3 for Windows* (Farrugia, 1997 ▶); software used to prepare material for publication: *SHELXL97* and *PLATON* (Spek, 2009 ▶).

## Supplementary Material

Crystal structure: contains datablocks I, global. DOI: 10.1107/S1600536810054036/bx2338sup1.cif

Structure factors: contains datablocks I. DOI: 10.1107/S1600536810054036/bx2338Isup2.hkl

Additional supplementary materials:  crystallographic information; 3D view; checkCIF report

## Figures and Tables

**Fig. 1.**
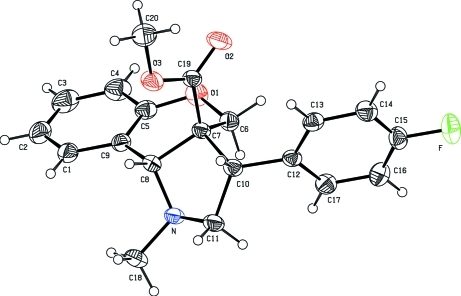
The molecular structure of the title compound, with displacement ellipsoids drawn at the 30% probability level.

## supplementary crystallographic information

### Comment

Chromanone derivatives are used as a varsatile intermediate in the synthesis of natural products calonlide(A) and inophyllum(B), (Ellis *et al.*, 1977) which have been suggested to have activity against anti-human immuno deficiency virus type1 (HIV-1) (Hussain *et al.*, 1986). Chromanone derivatives dilate the heart and act as remedies for angina pectoris (Hasegnaida, 1967). Spiropyrrolidine derivatives are often used in the synthesis of biologically active compounds. Spiro ring system is also very interesting from a biogenetic point of view. Synthetic pyrrolidine derivatives have activity against the aldose reductase enzyme, which controls influenza. Pyrrolidine derivatives possess anti-influenza (Stylianakis *et al.*, 2003) and anti- convulsant (Obniska *et al.*, 2002) activity. We report here the crystal structure of the title compound, Fig.1. The C—C, N—C, C—O bond lengths in the structure are close to those found in the related structures (Usha *et al.*, 2003; Abdul Ajees *et al.*, 2002). The sum of the angle around atom N (332°) indicates sp^3^ hybridization. The carboxylate group is perpendicular to the pyran ring [89.4 (1)°].The pyran ring adopts twist-half chair conformation with lowest asymmetry parameters (Nardelli, 1983) of Δ (C9—C5) =0.033 (1) and the pyrrole ring adopts half chair conformation Δs(C8) = 0.008 (1). The molecular conformation is stabilizaed by two weak C—H···N and C—H···O intramolecular interactions, Table 1. The crystal packing is stabilized by van der Waals forces.

### Experimental

A mixture of (*E*)-methyl2-((2-formylphenoxy) methyl)-3-(4-fluorophenyl)acrylate(2 mmol) and sarcosine (2 mmol) in acetonitrile (8 ml) was refluxed for 5h. After the completion of the reaction mixture was concentrated and the resulting crude mass was diluted with water (15 ml). The combined organic layer was washed with brine (2x10ml) and dried over anhydrous Na_2_SO_4._The organic layer was concentrated and purified by column chromatographyon silica gel (Acme 100–200 mesh), using ethyl acetate-hexanes (1:9) to afford the pure title of the compound as a colourless solid in 65% yield.

### Refinement

The H atoms were positioned geometrically and were treated as riding on their parent C atoms, with aromatic C—H = 0.93Å , methyl C-H = 0.96Å and methylene C—H = 0.97Å and with *U*_iso_ = 1.5U_eq_(C) for methyl H and 1.2U_eq_(*C*) for the remaining H atoms.

### Crystal data

**Table:**

C_20_H_20_FNO_3_	*F*(000) = 720
*M**_r_* = 341.37	*D*_x_ = 1.337 Mg m^−^^3^
Monoclinic, *P*2_1_/*c*	Mo *K*α radiation, λ = 0.71073 Å
Hall symbol: -P 2ybc	Cell parameters from 4237 reflections
*a* = 10.4519 (4) Å	θ = 2–28°
*b* = 20.6778 (8) Å	µ = 0.10 mm^−^^1^
*c* = 7.8508 (3) Å	*T* = 293 K
β = 91.535 (2)°	Block, colourless
*V* = 1696.12 (11) Å^3^	0.22 × 0.20 × 0.19 mm
*Z* = 4	

### Data collection

**Table:**

Bruker Kappa APEXII CCD diffractometer	2844 reflections with *I* > 2σ(*I*)
Radiation source: fine-focus sealed tube	*R*_int_ = 0.035
graphite	θ_max_ = 28.4°, θ_min_ = 2.0°
ω and φ scan	*h* = −13→13
16737 measured reflections	*k* = −27→27
4237 independent reflections	*l* = −10→10

### Refinement

**Table:**

Refinement on *F*^2^	Secondary atom site location: difference Fourier map
Least-squares matrix: full	Hydrogen site location: inferred from neighbouring sites
*R*[*F*^2^ > 2σ(*F*^2^)] = 0.045	H-atom parameters constrained
*wR*(*F*^2^) = 0.219	*w* = 1/[σ^2^(*F*_o_^2^) + (0.2*P*)^2^] where *P* = (*F*_o_^2^ + 2*F*_c_^2^)/3
*S* = 0.81	(Δ/σ)_max_ < 0.001
4237 reflections	Δρ_max_ = 0.24 e Å^−^^3^
227 parameters	Δρ_min_ = −0.18 e Å^−^^3^
0 restraints	Extinction correction: *SHELXL*, Fc^*^=kFc[1+0.001xFc^2^λ^3^/sin(2θ)]^-1/4^
0 constraints	Extinction coefficient: 0.013 (4)
Primary atom site location: structure-invariant direct methods	

### Special details

**Table:**

Geometry. All e.s.d.'s (except the e.s.d. in the dihedral angle between two l.s. planes) are estimated using the full covariance matrix. The cell e.s.d.'s are taken into account individually in the estimation of e.s.d.'s in distances, angles and torsion angles; correlations between e.s.d.'s in cell parameters are only used when they are defined by crystal symmetry. An approximate (isotropic) treatment of cell e.s.d.'s is used for estimating e.s.d.'s involving l.s. planes.
Refinement. Refinement of *F*^2^ against ALL reflections. The weighted *R*-factor *wR* and goodness of fit *S* are based on *F*^2^, conventional *R*-factors *R* are based on *F*, with *F* set to zero for negative *F*^2^. The threshold expression of *F*^2^ > σ(*F*^2^) is used only for calculating *R*-factors(gt) *etc*. and is not relevant to the choice of reflections for refinement. *R*-factors based on *F*^2^ are statistically about twice as large as those based on *F*, and *R*- factors based on ALL data will be even larger.

### Fractional atomic coordinates and isotropic or equivalent isotropic displacement parameters (Å^2^)

**Table:**

	*x*	*y*	*z*	*U*_iso_*/*U*_eq_	
C20	0.1861 (2)	0.22098 (11)	0.9717 (3)	0.0645 (6)	
H20A	0.1703	0.2651	0.9389	0.097*	
H20B	0.2338	0.2201	1.0780	0.097*	
H20C	0.1060	0.1990	0.9845	0.097*	
O1	0.53935 (13)	0.05675 (7)	0.87998 (17)	0.0559 (4)	
F	−0.01102 (13)	−0.15643 (6)	0.7317 (2)	0.0775 (5)	
C1	0.63732 (18)	0.20543 (10)	0.6667 (2)	0.0505 (5)	
H1	0.6080	0.2352	0.5858	0.061*	
C2	0.7536 (2)	0.21569 (12)	0.7493 (3)	0.0655 (6)	
H2	0.8025	0.2518	0.7236	0.079*	
C3	0.7969 (2)	0.17199 (14)	0.8701 (3)	0.0737 (7)	
H3	0.8757	0.1785	0.9253	0.088*	
C4	0.7248 (2)	0.11893 (12)	0.9099 (3)	0.0640 (6)	
H4	0.7545	0.0898	0.9922	0.077*	
C5	0.60721 (17)	0.10867 (9)	0.8271 (2)	0.0458 (4)	
C6	0.42920 (17)	0.03806 (8)	0.7786 (2)	0.0422 (4)	
H6A	0.3763	0.0094	0.8447	0.051*	
H6B	0.4569	0.0143	0.6796	0.051*	
C7	0.35015 (15)	0.09573 (8)	0.72016 (18)	0.0357 (4)	
C8	0.43498 (15)	0.14033 (8)	0.61491 (19)	0.0359 (4)	
H8	0.3916	0.1818	0.5955	0.043*	
C9	0.56300 (16)	0.15160 (8)	0.7018 (2)	0.0398 (4)	
C10	0.24295 (16)	0.07657 (8)	0.58563 (19)	0.0383 (4)	
H10	0.1776	0.1105	0.5884	0.046*	
C11	0.30986 (18)	0.08318 (10)	0.4144 (2)	0.0485 (5)	
H11A	0.3104	0.0420	0.3554	0.058*	
H11B	0.2657	0.1146	0.3425	0.058*	
C12	0.17597 (15)	0.01306 (8)	0.62225 (18)	0.0373 (4)	
C13	0.06850 (16)	0.01298 (9)	0.7231 (2)	0.0440 (4)	
H13	0.0382	0.0520	0.7649	0.053*	
C14	0.00570 (18)	−0.04361 (10)	0.7628 (2)	0.0515 (5)	
H14	−0.0650	−0.0433	0.8323	0.062*	
C15	0.05039 (18)	−0.09990 (9)	0.6970 (2)	0.0503 (5)	
C16	0.1548 (2)	−0.10261 (9)	0.5965 (3)	0.0540 (5)	
H16	0.1828	−0.1418	0.5532	0.065*	
C17	0.21785 (17)	−0.04557 (9)	0.5604 (2)	0.0475 (4)	
H17	0.2898	−0.0467	0.4932	0.057*	
C18	0.49406 (18)	0.14114 (10)	0.3144 (2)	0.0501 (5)	
H18A	0.4949	0.1146	0.2141	0.075*	
H18B	0.5799	0.1542	0.3444	0.075*	
H18C	0.4424	0.1788	0.2925	0.075*	
C19	0.28728 (16)	0.12722 (8)	0.87041 (19)	0.0397 (4)	
N	0.44108 (14)	0.10458 (7)	0.45389 (16)	0.0395 (4)	
O2	0.25895 (16)	0.09942 (7)	0.99746 (16)	0.0637 (5)	
O3	0.25839 (14)	0.18923 (6)	0.84246 (17)	0.0556 (4)	

### Atomic displacement parameters (Å^2^)

**Table:**

	*U*^11^	*U*^22^	*U*^33^	*U*^12^	*U*^13^	*U*^23^
C20	0.0666 (14)	0.0625 (13)	0.0655 (13)	0.0059 (10)	0.0216 (11)	−0.0122 (10)
O1	0.0514 (8)	0.0618 (8)	0.0535 (8)	−0.0039 (6)	−0.0194 (6)	0.0170 (6)
F	0.0686 (9)	0.0619 (8)	0.1021 (11)	−0.0257 (6)	0.0022 (8)	0.0114 (7)
C1	0.0503 (10)	0.0546 (11)	0.0470 (10)	−0.0087 (8)	0.0096 (8)	−0.0091 (8)
C2	0.0541 (12)	0.0807 (15)	0.0623 (13)	−0.0264 (11)	0.0128 (10)	−0.0206 (11)
C3	0.0476 (12)	0.111 (2)	0.0623 (14)	−0.0190 (13)	−0.0064 (10)	−0.0203 (13)
C4	0.0501 (12)	0.0916 (17)	0.0497 (11)	−0.0018 (11)	−0.0114 (9)	−0.0022 (10)
C5	0.0411 (9)	0.0571 (11)	0.0391 (9)	0.0006 (8)	−0.0026 (7)	−0.0023 (7)
C6	0.0451 (9)	0.0424 (9)	0.0387 (8)	−0.0015 (7)	−0.0065 (7)	0.0064 (6)
C7	0.0374 (8)	0.0414 (9)	0.0281 (7)	−0.0010 (6)	−0.0008 (6)	0.0035 (6)
C8	0.0375 (8)	0.0386 (8)	0.0316 (7)	0.0014 (6)	0.0028 (6)	0.0048 (6)
C9	0.0382 (9)	0.0447 (10)	0.0364 (8)	−0.0022 (7)	0.0028 (7)	−0.0052 (6)
C10	0.0396 (8)	0.0442 (9)	0.0309 (8)	−0.0015 (7)	−0.0031 (6)	0.0040 (6)
C11	0.0512 (10)	0.0646 (12)	0.0294 (8)	−0.0137 (9)	−0.0015 (7)	0.0043 (7)
C12	0.0343 (8)	0.0473 (10)	0.0300 (7)	−0.0023 (7)	−0.0021 (6)	0.0005 (6)
C13	0.0386 (9)	0.0537 (11)	0.0398 (9)	−0.0001 (7)	0.0023 (7)	−0.0068 (7)
C14	0.0385 (9)	0.0679 (13)	0.0481 (10)	−0.0096 (8)	0.0047 (8)	−0.0005 (8)
C15	0.0448 (10)	0.0505 (11)	0.0554 (11)	−0.0122 (8)	−0.0055 (8)	0.0064 (8)
C16	0.0533 (11)	0.0453 (11)	0.0632 (12)	0.0009 (8)	−0.0009 (9)	−0.0061 (8)
C17	0.0401 (9)	0.0540 (11)	0.0486 (10)	0.0000 (8)	0.0077 (7)	−0.0046 (8)
C18	0.0481 (10)	0.0679 (12)	0.0345 (8)	−0.0039 (9)	0.0060 (7)	0.0094 (8)
C19	0.0403 (9)	0.0481 (10)	0.0305 (8)	−0.0058 (7)	−0.0014 (6)	0.0015 (7)
N	0.0420 (8)	0.0486 (8)	0.0280 (6)	−0.0016 (6)	0.0018 (5)	0.0029 (5)
O2	0.0889 (12)	0.0667 (10)	0.0364 (7)	0.0026 (7)	0.0172 (7)	0.0078 (6)
O3	0.0688 (9)	0.0489 (8)	0.0502 (7)	0.0061 (6)	0.0222 (6)	0.0000 (6)

### Geometric parameters (Å, °)

**Table:**

C20—O3	1.440 (2)	C8—C9	1.504 (2)
C20—H20A	0.9600	C8—H8	0.9800
C20—H20B	0.9600	C10—C12	1.519 (2)
C20—H20C	0.9600	C10—C11	1.538 (2)
O1—C5	1.358 (2)	C10—H10	0.9800
O1—C6	1.435 (2)	C11—N	1.466 (2)
F—C15	1.364 (2)	C11—H11A	0.9700
C1—C2	1.379 (3)	C11—H11B	0.9700
C1—C9	1.389 (2)	C12—C17	1.382 (3)
C1—H1	0.9300	C12—C13	1.392 (2)
C2—C3	1.378 (4)	C13—C14	1.381 (3)
C2—H2	0.9300	C13—H13	0.9300
C3—C4	1.371 (4)	C14—C15	1.361 (3)
C3—H3	0.9300	C14—H14	0.9300
C4—C5	1.391 (3)	C15—C16	1.365 (3)
C4—H4	0.9300	C16—C17	1.384 (3)
C5—C9	1.395 (3)	C16—H16	0.9300
C6—C7	1.515 (2)	C17—H17	0.9300
C6—H6A	0.9700	C18—N	1.453 (2)
C6—H6B	0.9700	C18—H18A	0.9600
C7—C19	1.513 (2)	C18—H18B	0.9600
C7—C8	1.536 (2)	C18—H18C	0.9600
C7—C10	1.570 (2)	C19—O2	1.1953 (19)
C8—N	1.467 (2)	C19—O3	1.334 (2)
			
O3—C20—H20A	109.5	C12—C10—C11	117.71 (14)
O3—C20—H20B	109.5	C12—C10—C7	114.52 (12)
H20A—C20—H20B	109.5	C11—C10—C7	103.45 (12)
O3—C20—H20C	109.5	C12—C10—H10	106.8
H20A—C20—H20C	109.5	C11—C10—H10	106.8
H20B—C20—H20C	109.5	C7—C10—H10	106.8
C5—O1—C6	117.45 (13)	N—C11—C10	106.67 (12)
C2—C1—C9	121.4 (2)	N—C11—H11A	110.4
C2—C1—H1	119.3	C10—C11—H11A	110.4
C9—C1—H1	119.3	N—C11—H11B	110.4
C3—C2—C1	119.4 (2)	C10—C11—H11B	110.4
C3—C2—H2	120.3	H11A—C11—H11B	108.6
C1—C2—H2	120.3	C17—C12—C13	117.85 (16)
C2—C3—C4	120.6 (2)	C17—C12—C10	122.69 (15)
C2—C3—H3	119.7	C13—C12—C10	119.46 (15)
C4—C3—H3	119.7	C14—C13—C12	121.66 (17)
C3—C4—C5	119.9 (2)	C14—C13—H13	119.2
C3—C4—H4	120.0	C12—C13—H13	119.2
C5—C4—H4	120.0	C15—C14—C13	117.96 (16)
O1—C5—C4	116.08 (17)	C15—C14—H14	121.0
O1—C5—C9	123.58 (16)	C13—C14—H14	121.0
C4—C5—C9	120.30 (18)	C14—C15—C16	122.85 (17)
O1—C6—C7	112.27 (14)	C14—C15—F	119.26 (17)
O1—C6—H6A	109.1	C16—C15—F	117.89 (17)
C7—C6—H6A	109.1	C15—C16—C17	118.42 (17)
O1—C6—H6B	109.1	C15—C16—H16	120.8
C7—C6—H6B	109.1	C17—C16—H16	120.8
H6A—C6—H6B	107.9	C12—C17—C16	121.23 (17)
C19—C7—C6	110.33 (13)	C12—C17—H17	119.4
C19—C7—C8	115.48 (13)	C16—C17—H17	119.4
C6—C7—C8	108.52 (13)	N—C18—H18A	109.5
C19—C7—C10	108.43 (13)	N—C18—H18B	109.5
C6—C7—C10	112.26 (13)	H18A—C18—H18B	109.5
C8—C7—C10	101.62 (12)	N—C18—H18C	109.5
N—C8—C9	114.21 (13)	H18A—C18—H18C	109.5
N—C8—C7	101.54 (12)	H18B—C18—H18C	109.5
C9—C8—C7	111.63 (13)	O2—C19—O3	122.65 (16)
N—C8—H8	109.7	O2—C19—C7	124.48 (17)
C9—C8—H8	109.7	O3—C19—C7	112.74 (13)
C7—C8—H8	109.7	C18—N—C8	114.43 (14)
C1—C9—C5	118.24 (16)	C18—N—C11	111.78 (13)
C1—C9—C8	122.01 (16)	C8—N—C11	105.79 (13)
C5—C9—C8	119.69 (15)	C19—O3—C20	116.29 (15)
			
C9—C1—C2—C3	−0.5 (3)	C8—C7—C10—C11	26.42 (16)
C1—C2—C3—C4	−0.6 (4)	C12—C10—C11—N	−127.87 (15)
C2—C3—C4—C5	0.4 (4)	C7—C10—C11—N	−0.45 (18)
C6—O1—C5—C4	−169.32 (16)	C11—C10—C12—C17	31.4 (2)
C6—O1—C5—C9	12.9 (3)	C7—C10—C12—C17	−90.51 (19)
C3—C4—C5—O1	−176.91 (19)	C11—C10—C12—C13	−149.15 (15)
C3—C4—C5—C9	1.0 (3)	C7—C10—C12—C13	88.96 (18)
C5—O1—C6—C7	−42.6 (2)	C17—C12—C13—C14	0.8 (2)
O1—C6—C7—C19	−68.27 (17)	C10—C12—C13—C14	−178.74 (15)
O1—C6—C7—C8	59.16 (17)	C12—C13—C14—C15	−1.4 (3)
O1—C6—C7—C10	170.67 (13)	C13—C14—C15—C16	0.9 (3)
C19—C7—C8—N	−160.23 (13)	C13—C14—C15—F	−178.91 (17)
C6—C7—C8—N	75.34 (14)	C14—C15—C16—C17	0.1 (3)
C10—C7—C8—N	−43.13 (15)	F—C15—C16—C17	179.98 (17)
C19—C7—C8—C9	77.68 (16)	C13—C12—C17—C16	0.4 (3)
C6—C7—C8—C9	−46.75 (17)	C10—C12—C17—C16	179.85 (16)
C10—C7—C8—C9	−165.22 (13)	C15—C16—C17—C12	−0.8 (3)
C2—C1—C9—C5	1.8 (3)	C6—C7—C19—O2	−28.0 (2)
C2—C1—C9—C8	178.95 (16)	C8—C7—C19—O2	−151.46 (17)
O1—C5—C9—C1	175.64 (16)	C10—C7—C19—O2	95.34 (19)
C4—C5—C9—C1	−2.1 (3)	C6—C7—C19—O3	156.20 (14)
O1—C5—C9—C8	−1.5 (3)	C8—C7—C19—O3	32.72 (18)
C4—C5—C9—C8	−179.23 (16)	C10—C7—C19—O3	−80.49 (16)
N—C8—C9—C1	88.40 (19)	C9—C8—N—C18	−71.69 (18)
C7—C8—C9—C1	−157.13 (15)	C7—C8—N—C18	168.03 (13)
N—C8—C9—C5	−94.54 (18)	C9—C8—N—C11	164.82 (14)
C7—C8—C9—C5	19.9 (2)	C7—C8—N—C11	44.53 (15)
C19—C7—C10—C12	−82.09 (17)	C10—C11—N—C18	−152.67 (15)
C6—C7—C10—C12	40.05 (19)	C10—C11—N—C8	−27.52 (18)
C8—C7—C10—C12	155.80 (13)	O2—C19—O3—C20	−2.3 (3)
C19—C7—C10—C11	148.52 (14)	C7—C19—O3—C20	173.64 (16)
C6—C7—C10—C11	−89.33 (16)		

### Hydrogen-bond geometry (Å, °)

**Table:**

*D*—H···*A*	*D*—H	H···*A*	*D*···*A*	*D*—H···*A*
C6—H6B···N	0.97	2.58	2.902 (2)	100
C8—H8···O3	0.98	2.42	2.792 (2)	102
